# MicroRNA-181b-2 and MicroRNA-21-1 Negatively Regulate NF-κB and IRF3-Mediated Innate Immune Responses *via* Targeting TRIF in Teleost

**DOI:** 10.3389/fimmu.2021.734520

**Published:** 2021-12-09

**Authors:** Yuena Sun, Lei Zhang, Ling Hong, Weiwei Zheng, Junxia Cui, Xuezhu Liu, Tianjun Xu

**Affiliations:** ^1^ Laboratory of Fish Molecular Immunology, College of Fisheries and Life Science, Shanghai Ocean University, Shanghai, China; ^2^ Laboratory of Marine Biology and Biotechnology, Qingdao National Laboratory for Marine Science and Technology, Qingdao, China; ^3^ School of Medicine, Tongji University, Shanghai, China; ^4^ Laboratory of Fish Biogenetics & Immune Evolution, College of Marine Science, Zhejiang Ocean University, Zhoushan, China; ^5^ Key Laboratory of Exploration and Utilization of Aquatic Genetic Resources (Shanghai Ocean University), Ministry of Education, Shanghai, China; ^6^ National Pathogen Collection Center for Aquatic Animals, Shanghai Ocean University, Shanghai, China

**Keywords:** microRNA, TRIF, gene regulation, immune response, innate immune

## Abstract

Upon recognition of bacterial or viral components by Toll-like receptors (TLRs), cells could be activated to induce a series of reactions to produce inflammatory cytokines, type I interferon (IFN), and IFN stimulating genes (ISG). MicroRNAs (miRNAs) are an important regulatory molecules that are widely involved in the regulatory networks of mammalian inflammation and immune responses; however, in lower vertebrates, the regulatory network of miRNA-mediated immune responses is poorly understood. Here, we report two miRNAs form *Miichthys miiuy*, namely, miR-181b-2 and miR-21-1, that play a negative role in host antiviral and antibacterial immunity. We found that miR-181b-2 and miR-21-1 are abundantly expressed in gram-negative bacteria, as well as RNA rhabdovirus infection. Inducible miR-181b-2 and miR-21-1 suppress the production of inflammatory cytokines and type I IFN by targeting TRIF, thereby avoiding excessive inflammation. We further revealed that miR-181b-2 and miR-21-1 modulate antibacterial and antiviral immunity through the TRIF-mediated NF-κB and IRF3 signaling pathways. The overall results indicate that miR-181b-2 and miR-21-1 act as negative feedback regulators and participate in host antibacterial and antiviral immune responses; this finding could provide information for a deeper understanding of the resistance of lower vertebrates to the invasion of pathogens and to avoidance of excessive immunity.

## Introduction

Toll-like receptors (TLRs) play an important role in the activation of innate immunity by recognizing specific patterns of microbial components; the related signaling pathways consist of the MyD88-dependent pathway and the MyD88-independent pathway. In the MyD88-dependent pathway, MyD88 binds to TLRs through their C-terminal TIR domain as a connector molecule, after which its N-terminal dead domain activates the downstream IRAKs family. Following a series of transductions, it transmits TLRs-mediated signals to the cells, which ultimately leads to the activation and translocation of nuclear factor κB (NF-κB) (1, 2). Some studies have found that lipopolysaccharide (LPS) can activate TLR4 and NF-κB (3) in MyD88 knockout mice. Therefore, a MyD88-independent pathway may be involved in the process of TLR4 signal transduction. Subsequent studies found that LPS stimulation could activate interferon (INF) regulatory factor 3 (IRF3) in MyD88 knockout cells through the TLR4 pathway and promote IFN-induced gene expression. DsRNA could also activate NF-κB and IRF3 *via* the TLR3 pathway, which may follow the same MyD88-independent pathway as TLR4 for signal transduction (4). How TLR4 mediates the expression of type I IFN was previously unknown until TIR-domain-containing adapter-inducing interferon-β (TRIF) was discovered. According to the results of studies on TRIF-deficient mice induced by gene targeting or *N*-ethyl-*N*-nitrosourea mutagenesis, TRIF is essential for TLR4-mediated IFN expression (5). In fact, researchers had previously identified a TRIF in the TLR3 pathway by using yeast two-hybrid technology and named it TICAM-1; TICAM-1 is a TLR that contains the TIR domain and induces INF-β (interleukin 1) linker molecule (6, 7). Other studies have proven that no activation of NF-κB or up-regulation of inflammation-related genes occurs in TRIF and MyD88-deficient MBrC cells after LPS stimulation (8). Such studies confirm that TRIF plays an important role in the TLR4-mediated MyD88-independent pathway.

The signaling pathway connected by the TRIF is called the TRIF-dependent pathway (9). In mammals, the TRIF gene is composed of an N-terminal region, a TIR domain, a C-terminal region, an N-terminal region, and a tumor necrosis factor receptor-related factor (TRAF) family protein binding activator TBK1 (TANK-binding kinase-1), which mainly activates IFNs (10, 11). The C-terminal region of TRIF recruits receptor protein 1 and induces NF-κB activation (12). The TRIF gene has been cloned and identified in zebrafish, *Takifugu rubripes*, and *Ctenopharyngodon idella* (13–15). Studies have also revealed that the TRIF gene is involved in the antiviral immune response (15, 16). The TLR3 and TLR22 genes could recognize double-stranded RNA viruses and stimulate MBrC to trigger the transduction of the TRIF gene signaling pathway and activate MAPK, NF-κB, and IRF3/7, eventually inducing the production and release of inflammatory cytokines and IFN (17, 18). At present, an increasing body of evidence indicates that TRIF and its signaling pathways play an important role in the immune response and disease regulation. For example, researchers have found that the LPS-induced TLR4/TRIF pathway can activate the NLRP3 gene and that NLRP3 can activate caspase-1, which prompts cells to secrete IL-18 to the extracellular, thereby up-regulating Fasl and TNF-α and causing liver damage (19). Other studies have confirmed that damaged tissues can recruit myeloid cells for tissue repair through TLR3/TRIF-mediated cytokine secretion (20). Given the role of TRIF in disease, this protein has become a research focus in attempts to provide new insights and ideas for understanding the etiology of various diseases. The important role of TRIF in the immune response highlights the need to study the regulation and function of this protein. TRIF can be negatively regulated to suppress an organism’s immune response, prevent the organism from producing an excessive immune response, and maintain the immune system’s homeostasis. For example, studies have reported that IRF3 can negatively regulate TRIF-mediated NF-κB signaling pathways (21). Another study found that ADAM15 acts as a negative regulator of TRIF-mediated NF-κB and IFN-β reporter gene activity and acts as an anti-inflammatory molecule by disrupting TRIF-mediated TLR signaling (22). The present article focuses on the roles of miR-181b-2 and miR-21-1 in miiuy croaker (*Miichthys miiuy*) in regulating TRIF and the immune response.

MicroRNAs (miRNAs) are a type of endogenous single-stranded small-molecule RNAs with a length of approximately 21-23 bases. Single-stranded RNA precursors with a hairpin structure of approximately 70-90 bases pass through Dicer. It is produced after enzyme processing, which is different from siRNA (double-stranded) but closely related to siRNA. MiRNAs present a variety of essential regulatory functions in cells, such as cell proliferation, differentiation, and apoptosis (23, 24). The complex regulatory networks connected to miRNAs can either regulate the expression of multiple genes through one miRNA or finely regulate the expression of a gene through the combination of several miRNAs. For example, miR-8159-5p and miR-217-5p can target and regulate TLR1 expression in miiuy croaker (25). In addition, miR-3570 and miR-214 may negatively regulate the expression of the MyD88 gene in mackerel, thereby inhibiting the activation of NF-κB and the production of inflammatory cytokines (26, 27). Researchers speculate that one-third of all human genes are regulated by miRNAs. Mature miRNAs are not only conserved in various gene positions in different species but also show high homology in their sequences (28–30). Recent studies have found that miRNAs in some species exhibit transboundary regulation, which presents new ideas for miRNA research. For example, studies have found that miRNAs from *Cuscuta campestris* can target host mRNAs across species to regulate their gene expression, thereby indicating that these miRNAs could function as pathogenic factors and play a role in the process of *C. campestris* parasitism (31). In addition, plant miR-159 can inhibit the development of breast cancer, exogenous plant miR-168a can specifically target mammalian LDLRAP1, and the atypical microRNA2911 of honeysuckle directly targets influenza a virus (32–34). These studies demonstrated that the transboundary regulation of microRNAs. MiRNAs can be partially paired with the 3’untranslated region (UTR) of their target gene to regulate the expression of the target gene at the post-transcriptional level. Although the number of miRNAs involved in regulating the immune response in fish is increasing, they are still rare when compared with the miRNAs of two mammals. In this study, we found that miR-21-1 and miR-181b-2 can inhibit the activation of NF-κB, IRF3, and IFN by targeting TRIF, thereby suppressing the expression of inflammatory cytokines. Double luciferase reporter experiment confirmed this conclusion for the first time, that miR-21-1 and miR-181b-2 can target TRIF-3’UTR and inhibit its expression. We then demonstrated that miR-21-1 and miR-181b-2 can inhibit TRIF mRNA expression *via* qRT-PCR. Our findings were confirmed by Western blotting. MiR-21-1 and miR-181b-2 can inhibit the activation of NF-κB, IRF3, IFNα, and other reporter genes by targeting TRIF, which has been verified in EPC cells. Thus, studying its evolutionary history can help improve the understand of its mechanism and function.

## Materials and Methods

### Sample and Challenge

Miiuy croaker (~50 g) was obtained from Zhoushan Fisheries Research Institute, Zhejiang Province, China. Fish was acclimated in aerated seawater tanks at 25°C for six weeks before experiments. The challenge experiments were conducted as follows. Briefly, fish was respectively challenged with 100 µl of *V. anguillarum* (1.5 × 10^8^ CFU/ml), SCRV at a multiplicity of infection (MOI) of 5 or poly(I:C) (*In vivo*Gen, 1mg/ml) through intraperitoneal. Afterward, fishes were respectively sacrificed at different time points and the liver and spleen tissues were collected for RNA extraction. All animal experimental procedures were performed in accordance with the National Institutes of Health’s Guide for the Care and Use of Laboratory Animals, and the experimental protocols were approved by the Research Ethics Committee of Shanghai Ocean University (No. SHOU-DW-2018-047.

### Cell Culture and LPS/Poly(I:C) Stimulate

Miiuy croaker brain cell line (MBrC) were cultured in L-15 medium (HyClone) supplemented with 10% fetal bovine serum (FBS; Gibco), 100 U/ml penicillin, and 100μg/ml streptomycin at 26°C. MBrC line were prepared from the brain tissue of the miiuy croaker, and the isolation process is the same as previously reported (35). HEK293 cells were cultured in DMEM high glucose medium (HyClone) containing 10%FBS, 100 U/ml penicillin, and 100 μg/ml streptomycin at 37°C in 5% CO_2_. Epithelioma papulosum cyprini (EPC) cells were cultured in medium 199 (Invitrogen) supplemented with 10% FBS, 100 U/ml penicillin, and 100 mg/ml streptomycin at 28°C in 5% CO_2_. 24 hours after miR-21-1 and miR-181b-2 were transfected into MBrC cells, LPS/poly(I: C) was added to the cell culture medium by changing the medium. After 12 hours, cells were collected and total RNA was extracted and stored in -80°C standby.

### Prediction of MiR-21-1 and MiR-181b-2 Targeting Sites and Plasmid Construction

The miR-21-1 and miR-181b-2 targets were predicted using Targetscan (36), miRanda (37), and miRInspector (38) algorithms. To construct the TRIF-3’UTR reporter plasmid, miiuy croaker TRIF-3’UTR sequence was amplified using PCR and cloned into pmirGLO luciferase reporter plasmid, and the TRIF-3’UTR-MT mutant plasmid was constructed by using Mut Express II Rapid Mutagenesis Kit v2 (Vazyme Biotech). In order to construct TRIF expression plasmid, full-length coding sequence (CDS) and 3’UTR of TRIF gene in miiuy croaker were amplified by PCR with specific primers and inserted into pcDNA3.1 vector. The TAK1 3’UTR sequence was inserted into the mVenus-C1 vector to construct the green fluorescent protein (GFP) report plasmid. The pre-miR-21-1 and pre-miR-181b-2 sequence was amplified by PCR with specific primers and cloned into pcDNA3.1 vector. All plasmids were extracted by endotoxin-free plasmid DNA Miniprep kit (Tiangen) and confirmed by Sanger sequencing before using plasmids. The expression of protein was confirmed by Western blot analysis of full-length plasmid.

### Transfection and Virus Infection

Cells were seeded into 24-well plates and incubated overnight. Subsequently, MBrC cells were transfected with miR-21-1 or miR-181b-2 by using Lipofectamine 3000 (Invitrogen) according to the manufacturer’s protocols. 24 h after transfection, the MBrC were washed, the cells were infected with SCRV, and incubated at different times as indicated. Total RNA was extracted for quantitative real-time PCR (qPCR) analysis.

### MiRNA Mimic and Inhibitors

miR-21-1 or miR-181b-2 mimic (dsRNA oligonucleotides) and miR-21-1 or miR-181b-2 inhibitors (single stranded, chemically modified oligonucleotides), and control oligonucleotides were ordered from GenePharma (Shanghai, China). Sequences are as follows: miR-21-1 mimic, 5’-CAACAGCAGUCUGUAAGCUGGC-3’

(sense) and 5’-CAGCUUACAGACUGCUGUUGUU-3’ (antisense); miR-181b-2 mimic, 5’-AACAUUCAUUGCUGUCGCUGGG-3’

(sense) and 5’-CAGCGACAGCAAUGAAUGUUUU-3’ (antisense); negative control mimic, 5’-UUCUCCGAACGUGUCACGUTT-3’ (sense) and 5’-ACGUGACACGUUCGGAGAATT-3’ (antisense); miR-21-1 inhibitors, 5’-GCCAGCUUACAGACUGCUGUUG-3’ (chemically modified by 29-Ome) and miR-181b-2 inhibitors, 5’-CCCAGCGACAGCAAUGAAUGUU-3’ (chemically modified by 29-Ome) and negative control inhibitors, 5’-CAGUACUUUUGUGUAGUACAA- 3’. MBrC, EPC or HEK293 cells were transfected with 50-100 nM of each oligonucleotide.

### RNA Interference

The TRIF-specific small interfering RNA (si-TRIF) sequence were 5’-GAGACAACUACCUUGCUAGTT-3’(sense) and 5’-CUAGCAAGGUAGUUGUCUCTT-3’ (antisense). The scrambled control RNA sequences were 5’-UUCUCCGAACGUGUCACGUTT-3’ (sense) and 5’-ACGUGACACGUUCGGAGAATT-3’ (antisense). 100nM of each small interfering RNA (siRNA) were transfected into MBrC for up to 48 h and then stimulated with poly(I:C) or SCRV.

### Real-Time Quantitative PCR

We have used Lipofectamine™ RNAiMAX (Invitrogen) transfect miR-21-1 and miR-181b-2 mimics or miR-21-1 and miR-181b-2 inhibitors, NC mimic and NC inhibitor as negative control into MBrC cells. 24 hours after transfection, the MBrC cells were stimulated with LPS or poly(I:C) or SCRV, and then cells were collected with Trizol. Total RNA was isolated with Trizol reagent (Invitrogen) by following the Ultrapure RNA kit (CWBIO) instructions, and it was reverse transcribed into cDNA according to the HiScript II Q RT SuperMix (Vazyme Biotech) instructions. Then we designed specific RT primers to detect the expression of TRIF, TNF-α, Mx1 and IFN-2 genes, and β-actin was used as endogenous control to normalize the expression. The triple fluorescence intensity of each gene experimental group and control group was measured by Super Real PreMix Plus (Tian Gen) reagent and 7500 qRT-PCR system (Applied Biosystems, USA). At the end of the determination, the results of curve analysis were analyzed to determine the specificity of the target. For each sample, all amplification reactions were performed in triplicate. The sequences of all mRNA primers are listed in **Supplementary Table 1**.

### Dual-Luciferase Reporter Assays

The wild-type or mutant-type of the TRIF-3’UTR luciferase reporters were cotransfected with miR-21-1 or miR-181b-2 mimics or inhibitors, pre-miR-21-1 or pre-miR-181b-2 plasmid into EPC cells as well as HEK293 cells. Then TRIF-3’UTR luciferase activities were measured using the Dual-Luciferase Reporter Assay System (Promega). In order to exam TRIF functional regulation, EPC cells were cotransfected with NF-κB, IFN-2, IRF3, and IL-1β luciferase reporter gene, TRIF full-length expression plasmid, and phRL-TK Renilla luciferase plasmid, together with miR-21-1 or miR-181b-2, or pre-miR-21-1 and pre-miR-181b-2 for dual-luciferase reporter assays. Transfected 24 or 48 h later, cells were lysed for reporter activity testing using the Dual-Luciferase Reporter Assay System (Promega). All the results were obtained by three repeated independent experiments.

### Western Blotting

The miR-21-1 and miR-181b-2 mimics or pre-miR-21-1 and pre-miR-181b-2 plasmids and the TRIF full-length expression plasmid were cotransfected into HEK293 cells using Lipofectamine 3000 (Invitrogen) transfection reagent. After 48 h of transfection, the medium was aspirated, HEK293 cells were washed three times with cold phosphate-buffered saline (PBS), and the cellular lysates were generated by using 1×SDS-PAGE (10%) loading buffer. The disrupted cells were heated in a 95°C metal bath for 5 minutes, and the same number of samples were taken for SDS-PAGE electrophoresis and the protein was transferred to a PVDF membrane (Millipore) by semidry blotting (Bio-Rad Trans Blot Turbo System). The membranes were blocked with 5% BSA. Protein was blotted with different Abs. The Ab against anti-Flag and anti-Tubulin mAb were diluted at 1:2000 (Sigma-Aldrich), and HRP-conjugated anti-mouse IgG (Abbkine) was diluted at 1:5000. The results were representative of three independent experiments. Proteins were detected by using BeyoECL Plus (Beyotime) and digital imaging was performed using a cold CCD camera.

### Statistical Analysis

Data are expressed as the mean ± SD from at least three independent triplicated experiments. Student’s t-test was used to evaluate the data. The relative gene expression data was acquired using the 2 ^-ΔΔCT^ method and comparisons between groups were analyzed by one-way analysis of variance (ANOVA) followed by Duncan’s multiple comparison tests (39). A value of *p*< 0.05 was considered significant.

## Results

### LPS, *V. anguillarum* or Poly(I:C) Stimulation and SCRV Infection Significantly Upregulated miR-21-1 and miR-181b-2 Expression

**Figure 1:**
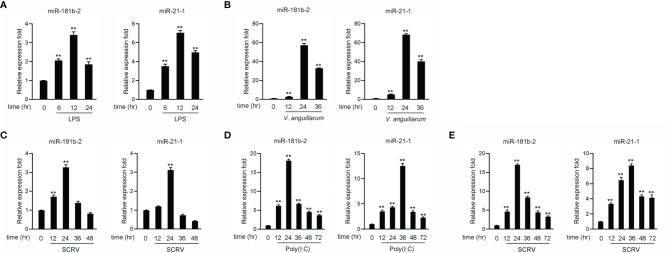
The expression analysis of miR-21-1 and miR-181b-2 following bacterial infection and virus injection. **(A)** The expression of miR-21-1 and miR-181b-2 in LPS-stimulation MBrC cells. **(B)** The expression of miR-21-1 and miR-181b-2 in miiuy croaker liver samples after *V. anguillarum* infection. **(C)** The expression of miR-21-1 and miR-181b-2 in the MBrC cells of SCRV injection. **(D)** The expression of miR-21-1 and miR-181b-2 in miiuy croaker spleen after poly(I:C) stimulation. **(E)** The expression of miR-181b-2 and miR-21-1 in miiuy croaker spleen after SCRV injection. The expression of miR-181b-2 and miR-21-1 were measured by qRT-PCR and normalized to 5.8S rRNA. All data are presented as the means ± SE from three independent triplicate experiments. **p < 0.01, versus the controls.

Hundreds of miRNAs were expressed in poly(I:C)-treated miiuy croaker liver (40, 41) to investigate the host-encoded miRNAs that may potentially be involved in regulating TRIF and its signaling pathways in fish upon virus injection. A miRNA target prediction program was then used to predict candidate miRNAs that bind to TRIF by using the 3’-UTR of miiuy croaker TRIF (**Supplementary Table 2**). Combining our present findings with those reported in our previous work, we found that two miRNAs, namely, miR-21-1 and miR-181b-2, were upregulated under the stimulation of poly(I:C) (40, 41). We examined the expression levels of miR-21-1and miR-181b-2 in miiuy croaker brain and liver tissue samples after LPS (a gram-negative endotoxin) or *V. anguillarum* stimulation by using qRT-PCR. The results showed that the expression levels of miR-21-1 and miR-181b-2 peak after 12 h in brain samples stimulated by LPS (**Figure 1A**) and after 24 h in liver tissues stimulated by *V. anguillarum* (**Figure 1B**). We used poly(I:C) or SCRV as pathogens to stimulate miiuy croaker and MBrC cells to detect changes in miR-21-1 and miR-181b-2 expression levels. As shown in **Figures 1C, D**, the expression of miR-21-1 and miR-181b-2 peaked after 24 h in SCRV-injected MBrC cells. In addition, and the expression of miR-181b-2 peaked after 24 h while the expression of miR-21-1 peaked after 36 h in poly(I:C)-stimulated MBrC cells. Finally, we examined the expression of miR-21-1 in SCRV-stimulated spleen tissue. The results showed that the expression of miR-21-1 peaks after 36h while the expression of miR-181b-2 peaks after 24h (**Figure 1E**). These results indicate that miR-21-1 and miR-181b-2 are involved in the immune response induced by miiuy croaker-infected pathogens.

### MiR-21-1 and MiR-181b-2 Negatively Regulate the Expression of Inflammatory Response

**Figure 2:**
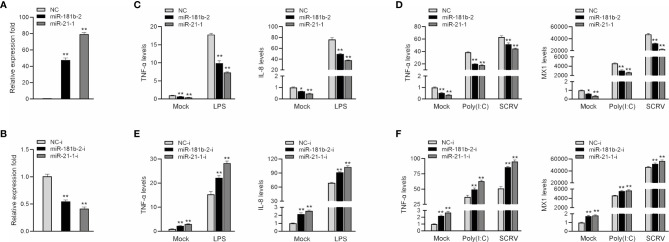
MiR-181b-2 and miR-21-1 inhibit LPS, poly(I:C) or SCRV induce inflammatory response. **(A, B)** We transfected miR-21-1 and miR-181b-2 mimic **(A)** and miR-21-1 and miR-181b-2 inhibitors or NC, inhibitor NC into macrophage. **(C, D)** We transfected miR-21-1 and miR-181b-2 mimic into MBrC cells, and NC was used as a negative control. 24 h after transfection, the cells of the experimental group were stimulated with LPS, or poly (I:C) stimulation or SCRV injection, and then total RNA was extracted from the cells. The mRNA expression of TNF-α, IL-8 and Mx1 were detected by qRT-PCR. **(E, F)** miR-21-1 or miR-181b-2 inhibitors were transfected into MBrC cells, and inhibitor NC was used as a negative control. 24 h after transfection, the cells were stimulated with LPS, poly (I:C) and SCRV stimulation, total RNA was extracted by trizol. The mRNA expression of TNF-α, IL-8 and Mx1 were analyzed by qRT-PCR. All data are presented as the means ± SE from three independent triplicate experiments. **p < 0.01, *p < 0.05 versus the controls.

To investigate the role of miR-21-1 and miR-181b-2 in the regulation of bacterium-induced inflammation, we examined the effect of miR-21-1 and miR-181b-2 mimics or inhibitors on the expression of inflammatory cytokines or antiviral effectors, such as IL-8, Mx1, and TNF-α, following LPS or poly(I:C) stimulation or SCRV injection. First, we examined the effects of synthetic miR-21-1 and miR-181b-2 mimics and inhibitors on the expression of the miRNAs. As shown in **Figures 2A, B**, after the transfection of synthetic miR-21-1 and miR-181b-2 mimics, the expression of miR-21-1 and miR-181b-2 significantly increased (**Figure 2A**). After the transfection of synthetic miR-21-1 and miR-181b-2 inhibitors, the expression of miR-21-1 and miR-181b-2 significantly decreased (**Figure 2B**). Next, we explored whether the overexpression of miR-21-1 and miR-181b-2 can affects the expression of inflammatory cytokines after LPS or poly(I:C) treatment, or SCRV injection. NC, the miR-21-1 and miR-181b-2 mimics, or miR-217 inhibitor were transfected into miiuy croaker MBrC cells prior to LPS or poly(I:C) stimulation or SCRV injection and analysis was performed 24 h later. The results showed that the overexpression of miR-21-1 or miR-181b-2 significantly inhibits the expression of TNF-α and IL-8 cytokines after LPS stimulation compared with NC (compared with NC; **Figure 2C**). The expression of TNF-α and MX1 was also inhibited after poly(I:C) and SCRV treatment (compared with NC; **Figure 2D**). On the contrary, the expression of TNF-α and IL-8 cytokines increased after transfection of miR-21-1 or miR-181b-2 inhibitor after LPS stimulation (compared with NC; **Figure 2E**), and the expression of TNF-α and MX1 were also increased after poly(I:C) or SCRV treatment (compared with NC; **Figure 2F**). The above data suggest that miR-21-1 and miR-181b-2 and their inhibitors can significantly increase or decrease, respectively, the expression levels of these proteins. Additionally, miR-21-1 and miR-181b-2 inhibited the production of inflammatory cytokines such as TNF-α, IL-8, and MX1 in response to LPS, poly(I:C), or SCRV stimulation, thus confirming the regulatory effect of miR-21-1 and miR-181b-2 on the immune system of miiuy croaker.

### TRIF Is a Direct Target Gene of MiR-21-1 and MiR-181b-2

**Figure 3:**
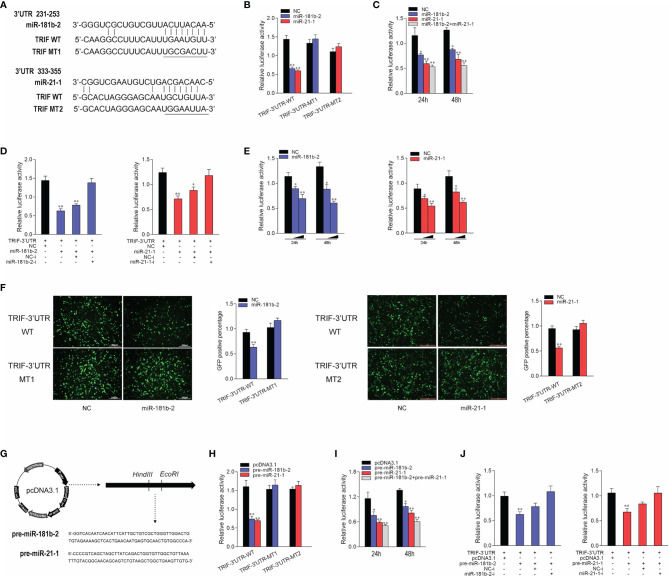
Miiuy croaker TRIF is a directly target of miR-21-1 and miR-181b-2. **(A)** Sequence alignment of the 3’UTR of TRIF and its binding sites. **(B)** TRIF-3’UTR-WT or TRIF-3’UTR–MT1, TRIF-3’UTR–MT2 was cotransfected with miR-21-1 or miR-181b-2 into HEK293 cells with NC as a negative control, and cells were harvested after 24 h to detect luciferase activity. **(C)** MiR-21-1 and miR-181b-2 was transfection with TRIF-3’UTR into HEK293 cells, 24 h later, to detect and normalize luciferase activity to *Renilla* luciferase activity. **(D)** Cotransfect TRIF-3’UTR with miR-21-1 or miR-181b-2 mimics or inhibitors into HEK293 cells, NC or NC inhibitors as negative control, and harvest cells 24 h later, to detect and normalize luciferase activity to *Renilla* luciferase activity. **(E)** TRIF-3’UTR was cotransfected with miR-21-1 or miR-181b-2 into HEK293 cells, with NC as negative control, and concentration gradients and time gradients were set respectively. **(F)** The wild-type or mutant mVenus-TRIF-3’UTR and miR-21-1 or miR-181b-2 were cotransfected into HEK293 cells. The right histogram is the percentage of GFP positive under excitation light. **(G)** Pre-miR-21-1 and pre-miR-181b-2 sequence was inserted into pcDNA3.1 vector using HindIII and EcoRI restriction sites to construct a pre-miR-21-1 or miR-181b-2 expression plasmid. **(H)** TRIF-3’UTR-WT or TRIF-3’UTR-MT1, TRIF-3’UTR-MT2 was cotransfected with pre-miR-21-1 or pre-miR-181b-2 plasmids into HEK293 cells with pcDNA3.1 as a negative control, and cells were harvested after 24 h to detect luciferase activity. **(I)** Pre-miR-21-1 and pre-miR-181b-2 was transfection with TRIF-3’UTR into HEK293 cells, 24 h later, to detect and normalize luciferase activity to *Renilla* luciferase activity. **(J)** Cotransfect TRIF-3’UTR with pre-miR-21-1 or pre-miR-181b-2 and miR-21-1 and miR-181b-2 inhibitors into HEK293 cells, pcDNA3.1 and NC inhibitors as negative control, and harvest cells 24 h later, to detect and normalize luciferase activity to *Renilla* luciferase activity. All data are presented as the means ± SE from three independent triplicate experiments. **p < 0.01, *p < 0.05 versus the controls.

To prove that TRIF is a potential target of miR-21-1 and miR-181b-2, we performed predictive analysis. TRIF appeared to harbor a target sequence for miR-181b-2 at nucleotides 231-253 of its 3’UTRs and another target sequence for miR-21-1 at nucleotides 333-355 of its 3’UTRs (**Figure 3A**). To obtain direct evidence that TRIF-3’UTR is a target of miR-181b-2 and miR-21-1, we cloned the miiuy croaker TRIF-3’UTR sequence into the pmri-GLO luciferase report vector and changed the miR-21-1 and miR-181b-2 binding sites to construct a TRIF-3’UTR mutant. Then, we co-transfected miR-21-1 and miR-181b-2 mimics with TRIF-3’UTR or the TRIF-3’UTR mutant into HEK293 cells; here, NC was used as the negative control. After 48 hours later, the luciferase activity was detected in the collected cells. The results showed that the miR-21-1 and miR-181b-2 mimics could decrease the luciferase activity of TRIF-3’UTR-WT, but had no such effect on the mutant TRIF-3’UTR-MT (**Figure 3B**). When HEK293 cells were cotransfected with the same amount of miR-21-1 and miR-181b-2 mimics and miR-21-1 and miR-181b-2 inhibitors, the downregulation of TRIF-3’UTR was inhibited by the mimics (**Figure 3C**). In addition, the combined effect of miR-21-1 and miR-181b-2 was better than that of either miRNA alone (**Figure 3D**). We also found that the miR-21-1 and miR-181b-2 mimics could inhibit TRIF-3’UTR luciferase activity in a dose-dependent and time-dependent manner (**Figure 3E**). When we cloned the TRIF-3’UTR into the mVenus-C1 vector and cotransfected it into HEK293 cells with the miR-21-1 and miR-181b-2 mimic, miR-21-1 and miR-181b-2 mimic down-regulated the expression of TRIF-3’UTR-GFP (WT), did not change the expression of TRIF-3’UTR-GFP (MT) (**Figure 3F**).

The pre-miR-21-1 and pre-miR-181b-2 sequences of miiuy croaker were cloned into the pcDNA3.1 vector to construct pre-miR-21-1 and pre-miR-181b-2 plasmids (**Figure 3G**). We cotransfected pre-miR-21-1 and pre-miR-181b-2 plasmids with TRIF-3’UTR or the TRIF-3’UTR mutant into HEK293 cells, and used pcDNA3.1 as the negative control. The results showed that pre-miR-21-1 and pre-miR-181b-2 plasmids could decrease the luciferase activity of TRIF-3’UTR-WT but had no effect on the mutant TRIF-3’UTR-MT (**Figure 3H**). In addition, we found that the miR-21-1 and miR-181b-2 mimics inhibited TRIF-3’UTR luciferase activity in a dose- and time-dependent manner (**Figure 3I**). When HEK293 cells were cotransfected with the same amount of pre-miR-21-1 or pre-miR-181b-2 plasmids and miR-21-1 or miR-181b-2 inhibitors, the downregulation of TRIF-3’UTR was inhibited. Moreover, the combined effect of pre-miR-21-1 and pre-miR-181b-2 was better than that of either pre-miRNA alone (**Figure 3J**). These results show that exogenous miR-21-1 and miR-181b-2 mimics could inhibit the expression of TRIF-3’UTR, and that TRIF is a new target of miR-21-1 and miR-181b-2.

### MiR-21-1 and MiR-181b-2 Regulate TRIF Expression at the Post-Transcriptional Level

**Figure 4:**
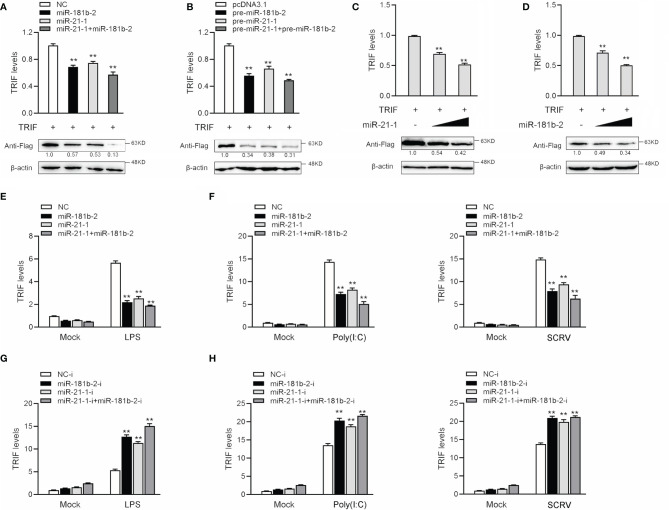
MiR-21-1and miR-181b-2 inhibits the expression of TRIF at post-transcriptional level. **(A)** HEK293 cells were cotransfected with TRIF expression plasmid with NC, miR-21-1 or miR-181b-2. After 48 h, TRIF protein and mRNA levels were determined by Western blotting and qRT-PCR, respectively. **(B)** HEK293 cells were cotransfected with TRIF expression plasmid with pcDNA3.1, pre-miR-21-1 or pre-miR-181b-2. After 48 h, TRIF protein and mRNA levels were determined by Western blotting and qRT-PCR, respectively. **(C, D)** HEK293 cells were cotransfected with TRIF expression plasmid, along with NC, miR-21-1 **(C)** (0, 50, and 100 nM) or miR-181b-2 **(D)** (0, 50, and 100 nM) in a concentration gradient manner, and NC was used to control the same amounts of molecules for transfections. After 48 h, TRIF protein and mRNA levels were determined by western blotting and qRT-PCR, respectively. **(E)** We transfected miR-21-1 and miR-181b-2 into MBrC cells, NC as negative control, and stimulated with LPS for 6 h, and the TRIF mRNA expression were determined by qRT-PCR. **(F)** miR-21-1 and miR-181b-2 were transfected into MBrC cells, NC as negative control, and stimulated with poly(I:C) for 12 h or SCRV injection for 12 h, and the TRIF mRNA expression were determined by qRT-PCR. **(G)** We transfected miR-21-1-i and miR-181b-2-i into MBrC cells, NC-i as negative control, and stimulated with LPS for 6 h, and the TRIF mRNA expression were determined by qRT-PCR. **(H)** miR-21-1-i and miR-181b-2-i were transfected into MBrC cells, NC-i as negative control, and stimulated with poly(I:C) for 12 h or SCRV injection for 12 h, and the TRIF mRNA expression were determined by qRT-PCR. All data are presented as the means ± SE from three independent triplicate experiments. **p < 0.01, versus the controls.

MicroRNAs function by binding to the 3’UTR of target mRNAs to regulate gene expression at the posttranslational level. To verify whether miR-21-1 and miR-181b-2 inhibit TRIF protein expression at the posttranslational level, we designed and synthesized CDS region and 3’UTR region-specific primers of miiuy croaker TRIF and then cloned them into pcDNA3-Flag vector plasmids by PCR amplification to construct a TRIF expression plasmid. Then, we transfected miR-21-1 and miR-181b-2 with the TRIF expression plasmid into HEK293 cells and used qPCR and Western blotting to detect changes in TRIF expression after 48 h transfection. The results showed that miR-21-1 and miR-181b-2 significantly inhibit TRIF mRNA and protein expression and that the combined effect of the two miRNAs is more significant than that of either miRNA alone (**Figure 4A**). The TRIF expression plasmid was cotransfected with pre-miR-21-1 and miR-181b-2 into HEK293 cells, and results showed that the combined effect of pre-miR-21-1 and pre-miR-181b-2 was stronger than that of either pre-miRNA alone (**Figure 4B**). This finding further shows that endogenous miR-21-1 and miR-181b-2 coregulate the expression of TRIF. We found that miR-21-1 and miR-181b-2 mimics inhibit TRIF mRNA and protein expression in a dose-dependent manner (**Figures 4C, D**).

To assess whether miR-181b-2 and miR-21-1 could also regulate the expression of TRIF at the mRNA level, we transfected the miRNA mimic, inhibitors, or NC into MBrC cells for 48 h and then stimulated the cells with LPS. The results shown in **Figures 4E, F** indicated that miR-181b-2 and miR-21-1 mimics could reduce the mRNA expression of TRIF following LPS stimulation; by contrast, miR-181b-2 and miR-21-1 inhibitors increased the mRNA expression of TRIF. The miRNA mimics, inhibitors, or NC were transfected into MBrC cells for 48 h, after which the cells were stimulated with poly(I:C) or SCRV. The results shown in **Figures 4G, H** indicate that miR-181b-2 and miR-21-1 mimics could reduce the mRNA expression of TRIF following poly(I:C) and SCRV treatment; by contrast, miR-181b-2 and miR-21-1 inhibitors increased the mRNA expression of TRIF. These results demonstrate that miR-181b-2 and miR-21-1 directly target TRIF during LPS or poly(I:C) stimulation or SCRV injection.

### MiR-21-1 and MiR-181b-2 Regulate the TRIF-Activated NF-κb Signaling Pathway

**Figure 5:**
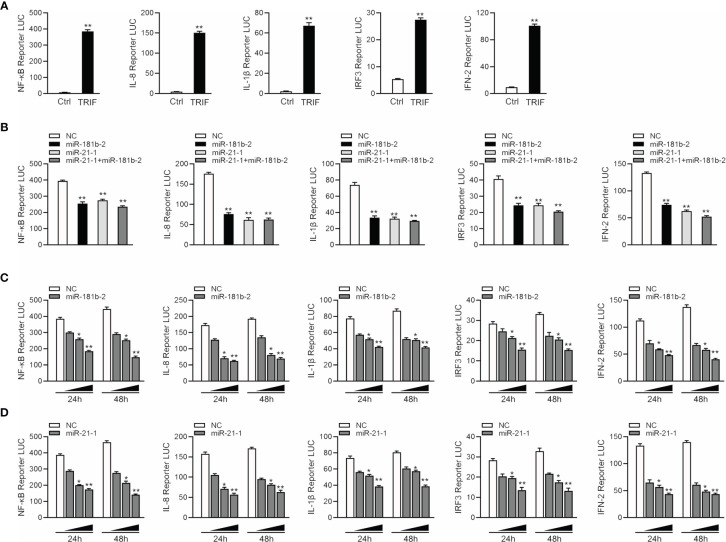
MiR-21-1 or miR-181b-2 regulates NF-κB signaling pathway by regulating TRIF. **(A)** TRIF expression plasmid and NF-κB, IL-8, IL1β, IFN-2, IRF3 with PhRL-TK luciferase reporter gene vector were cotransfection into EPC cells, mock is TRIF negative control, 48 h later detection of luciferase activity. **(B)** We cotransfect miR-21-1 and miR-181b-2 and TRIF expression plasmid with NF-κB, IL-8, IL1β, IFN-2 and IRF3 reporter gene and PhRL-TK luciferase reporter vector into EPC cells. After 24 h, cells were collected, and the firefly fluorescence intensity was measured. **(C, D)** miR-181b-2 (0, 25, 50, and 100 nM) **(C)** and miR-21-1 (0, 25, 50, and 100 nM) **(D)** along with NC (100, 75, 50, and 0 nM) and TRIF expression plasmid with NF-κB, IL-8, IL1β, IFN-2 and IFN-3 reporter gene and PhRL-TK luciferase reporter vector were transfected into EPC cells, and designed concentration-gradient. After 24 or 48 h, cells were collected and the firefly fluorescence intensity was measured, and normalization was performed according to the fluorescence activity of the *Renilla*. All data are presented as the means ± SE from three independent triplicate experiments. **p < 0.01, *p < 0.05 versus the controls.

Given that miR-144 and miR-217 modulate the expression of inflammatory cytokines (42), we examined whether TRIF can activate NF-κB and other signaling pathway genes. We co-transfected TRIF; NF-κB, IL-8, IL1β, IRF3 and IFN-2 reporter genes; and phRL-TK *Renilla* luciferase reporter genes into EPC cells. As shown in **Figure 5A**, TRIF could significantly activate the luciferase activity of reporter genes such as NF-κB, IL-8, IL1β, IRF3, and IFN-2. We cotransfected miR-21-1 and miR-181b-2 with TRIF and the reporter genes into EPC cells to verify whether the miRNAs can inhibit the activation of TRIF on these genes. The results showed that miR-21-1 and miR-181b-2 could significantly inhibit the expression of some TRIF-activated reporter genes, such as NF-κB, IRF3, and IFN-2, and that the combined effect of miR-21-1 and miR-181b-2 was better than that of either miRNA alone (**Figure 5B**). In addition, we found that miR-21-1 and miR-181b-2 mimics inhibit TRIF-activated reporter genes in a dose-dependent manner 24 and 48 h posttransfection (**Figures 5C, D**). We further verified the regulation of TRIF by miR-21-1 and miR-181b-2. Specifically, miR-21-1 and miR-181b-2 could regulate TRIF-activated NF-κB or IRF3 signaling pathway genes.

### TRIF Knockdown Inhibits Antiviral and Bacterial Inflammation Responses

**Figure 6:**
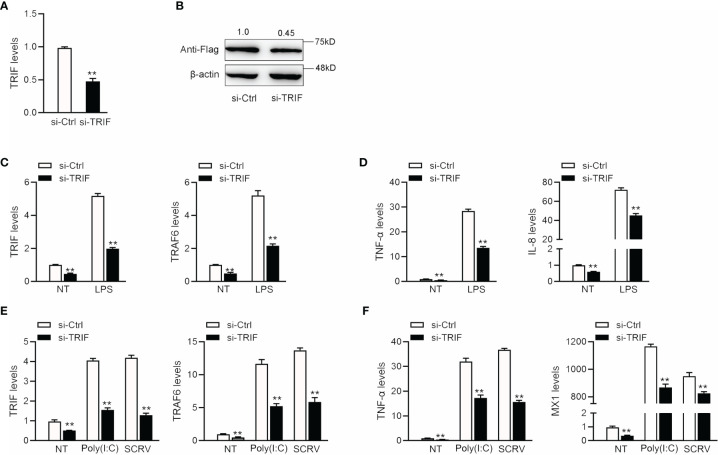
The expression levels of inflammatory cytokines after TRIF interference. **(A)** si-TRIF or si-NC were transfected into miiuy croaker MBrC cells, after 48 h, TRIF mRNA expression was measured. **(B)** TRIF expression plasmids, si-TRIF and si-NC were transfected into HEK293 cells, after 48 h, TRIF protein expression was measured by western blotting. **(C)** si-TRIF or si-NC were transfected into MBrC cells with LPS stimulation, after 48 h, TRIF and TRAF6 mRNA expression were measured by qRT-PCR. **(D)** si-TRIF or si-NC were transfected into MBrC cells with LPS stimulation, after 48 h, TNF-α and IL-8 mRNA expression were measured by qRT-PCR. **(E)** We transfected si-TRIF or si-NC into miiuy croaker MBrC cells with poly(I:C) stimulation or SCRV injection, TRIF and TRAF6 mRNA expression were tested by qRT-PCR. **(F)** We transfected si-TRIF or si-NC into miiuy croaker MBrC cells with poly(I:C) stimulation or SCRV injection, TNF-α and IL-8 mRNA expression were tested by qRT-PCR. All data are presented as the means ± SE from three independent triplicate experiments. **p < 0.01 versus the controls.

To confirm the effect of TRIF on antiviral and antibacterial inflammatory responses, we examined changes in inflammatory cytokine and antiviral gene expression after poly(I: C), SCRV, or LPS treatment. We designed a specific miiuy croaker TRIF siRNA (si-TRIF) and then transfected this molecule into MBrC cells. Results showed that si-TRIF could knock-down the expression of TRIF mRNA (**Figure 6A**). We cotransfected the TRIF expression plasmid and si-TRIF into HEK293 cells to verify the knockdown effect of si-TRIF. As shown in **Figure 6B**, si-TRIF inhibited the expression of TRIF protein. We transfected si-TRIF into miiuy croaker MBrC cells and used qRT-PCR to verify that whether TRIF regulates inflammatory cytokines. The results showed that after normal or LPS stimulation, siRNA could knock down the expression level of TRIF and suppress the expression of TRIF and its downstream TRAF6 gene (**Figure 6C**), thereby further downregulating the expression of TNF-α and IL-8 (**Figure 6D**). We verified whether knockdown TRIF under poly (I:C) stimulation and SCRV injection shows the same effect. Si-TRIF was transfected into MBrC cells and then treated with poly(I:C) or SCRV 24 h later. The results showed that si-TRIF consistently reduces the expression of TRIF mRNA, thereby inhibiting the expression of TRAF6 (**Figure 6E**), and inhibits the expression of TNF-α and MX1 (**Figure 6F**). This finding indicates that TRIF plays an important role in upstream and downstream genes. SiRNA can reduce the expression of TRIF, and miR-21-1 and miR-181b-2 have the same function as si-TRIF.

### MiR-21-1 and MiR-181b-2 Regulate the Expression of Some Components of the TRIF Signaling Cascade

**Figure 7:**
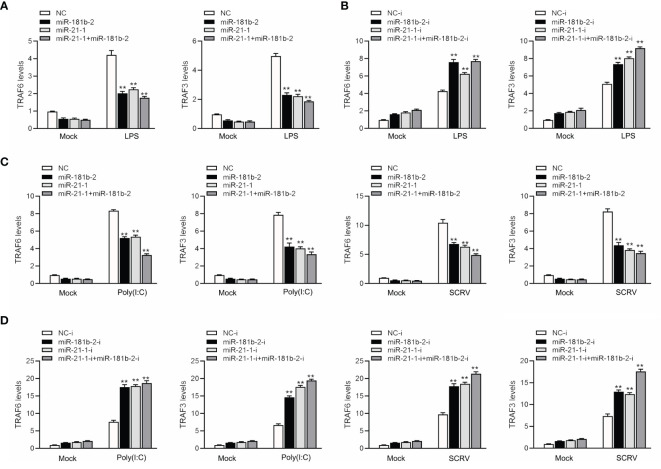
MiR-181b-2 and miR-21-1 regulates the expression of components of the TRIF signaling cascade. **(A, B)** We transfected NC, miR-181b-2 and miR-21-1 or NC-i, miR-181b-2-i and miR-21-1-i into MBrC cells for 48 h, then stimulated LPS for 6 h. Afterwards, the expression levels TRAF6 and TRAF3 were determined and normalized to β-actin. **(C, D)** We transfected NC, miR-181b-2 and miR-21-1 or NC-i, miR-181b-2-i and miR-21-1-i into MBrC cells for 48 h, then poly(I:C) stimulated and SCRV injection. Afterwards, the expression levels TRAF6 and TRAF3 were determined and normalized to β-actin. All data are presented as the means ± SE from three independent triplicate experiments. **p < 0.01, versus the controls.

To study the TRIF-induced signaling pathway in fish and examine whether miR-21-1 and miR-181b-2 participate in modulating TRIF-induced NF-κB signaling pathways, we transfected miR-21-1 or miR-181b-2 mimics or the NC mimic into MBrC cells, and then challenged the cells with LPS, poly(I:C), or SCRV. We harvested the cells 48 h later, extracted their RNA, and detected the expression of tumor necrosis factor-related factors 3 (TRAF3) and 6 (TRAF6) by qRT-PCR. As shown in **Figures 7A, B**, miR-21-1 and miR-181b-2 significantly inhibited TRAF3 and TRAF6 expression under mock, LPS stimulation. Moreover, the expressions of TRAF3 and TRAF6 were obviously decreased following miR-21-1 and miR-181b-2 mimic overexpression. We transfected miR-21-1 or miR-181b-2 inhibitors or NC-inhibitors into MBrC cells, and then challenged the cells with poly(I:C) or SCRV. Whereas they were significantly increased by miR-21-1 and miR-181b-2 inhibitors treatment in MBrC cells upon poly(I:C) or SCRV treatment (**Figures 7C, D**). MiR-21-1 and miR-181b-2 inhibited the expression of TRIF and its pathway gene TRAF6 under poly(I:C) stimulation. The combination of miR-21-1 and miR-181b-2 demonstrated more significant effects than either miRNA alone.

## Discussion

The innate immune system enables the body to produce a rapid and effective immune response to invading microorganisms. The key to ensuring this rapid response is that the body has a good recognition system. TLR recognition proteins are found in invading microorganisms; they stimulate intracellular signal transduction pathways and eventually produce a variety of immune responses. After a pathogen stimulates the body, all TLRs in the body stimulate the secretion of cytokines in the cells to initiate and promote the occurrence and development of inflammation. The stimulation of TLR3 and TLR4 causes the secretion of IFN-β through a single signal. This protein finally activates a series of genes, and its products help clear the pathogen. TLR3 can recognize double-stranded RNA and other molecules with similar structures, so it plays an important role in virus clearance. TLR4 is activated by the specific bacterial wall component LPS. The study of this pathway has been very clear, but it cannot explain all of the body’s responses to bacteria and viruses, especially IFN-2 production. A TRIF protein was recently reported to be a key component of this pathway (43, 44). TRIF is a new member of the TLRs signal transduction pathway. Research on this protein has gradually deepened, and the importance of TRIF-dependent signaling pathways to the body’s immunity is now better understood. Recent studies have found that TRAF3 and TRAF6 interact with the binding motif present in the N-terminal portion of TRIF through its region. Disrupting the TRAF9-binding motif of TRIF prevents it from connecting to TRAF6, resulting in the reduced activation of the NF-κB-dependent pathway induced by TRIF without affecting the IFN-β promoter. TBK1 is also connected to the N-terminal region of TRIF, and this link requires TBK1 kinase activation and TRIF phosphorylation. Because the binding sites of TRIF6 and TBK1 on TRIF are close to each other, TRAF6 may be able to physically prevent the connection of TBK1 and TRIF physically. Studies have found that IκB kinase I and TRAFs-related NF-κB activating molecule-binding kinase 1can be used as IRF3 kinases that are linked not only to IRF3 but also to TRIF (45, 46). The above results indicate that TRIF, TRAF6 and TBK1 are linked to activating two different transcription factors, NF-κB, and IRF3, respectively.

**Figure 8:**
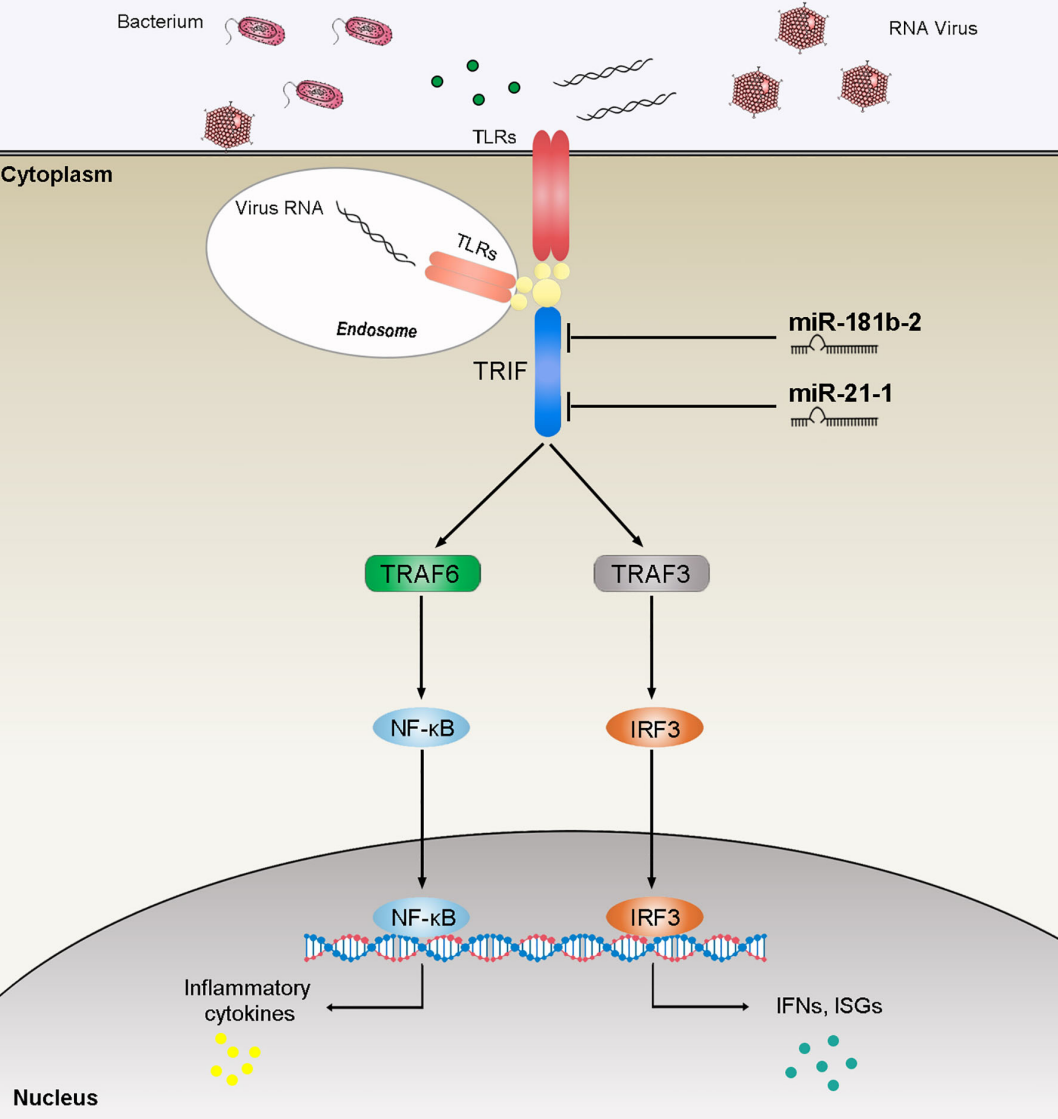
The speculated mechanism model miR-21-1 and miR-181b-2 negatively regulates NF-κB and IRF3 pathway by targeting TRIF. MiR-21-1 and miR-181b-2 target TRIF and represses TRIF-mediated innate immune responses.

TRIF, as an important linker molecule, plays an extremely important role in the bacterial and viral immune pathways. Recent research on the function and mechanism of TRIF has deepened our understanding of this molecule. We can speculate on the TRIF signal transduction pathways on the basis of previous experience (**Figure 8**). The virus invades cells and is recognized by TLRs. TLRs transduce signals to the linker molecule TRIF, which is divided into two pathways for the next level of transmission. One signal is transmitted to TBK1 and eventually triggers IRF3 activation. Another signal is transmitted to TRAF3 or TRAF6, which, in turn, triggers downstream NF-κB activation. These two pathways eventually promote the release of inflammatory cytokines. Research on the regulation of TRIF is important. For example, as the fifth identified TIR domain-containing adaptor protein SARM, it can specifically block the TRIF-mediated activation of transcription factors but has no effect on the MyD88 or non-TLR signaling pathways, such as the TNF and RIG-I pathways (47). At present, the regulatory mechanism of SARM is not very clear. The combination of SARM and TRIF is believed to block the recruitment of downstream signaling proteins by TRIF or recruit other unknown proteins that inhibit the TRIF pathway through the SAM domain. A series of studies have shown that certain proteins have a negative regulatory effect on TRIF. The SRC homology2 (SH2)-domain-containing protein tyrosine phosphatase 2 can also negatively regulate the production of cytokines and IFNs induced by the TRIF pathway of TLR3 and TLR4 without affecting the MyD88 pathway. The binding of TBK1 prevents it from affecting the activation of downstream pathways (48). The recently discovered TRIF negative regulatory protein TAG plays a role in the TLR4-mediated TRIF pathway (49). TAG, as a splicing mutant of TRAM, can replace the binding of TRIF and TRAM, and, therefore, specifically inhibits the TRIF pathway activated by TLR4. In addition, several studies have reported the involvement of miRNAs in TRIF-mediated signaling. The overexpression of cardiac miR-34a modulates TRIF-mediated cardiac remodeling in aldosterone-treated mice (50). In this article, we studied miR-21-1 and miR-181b-2 as negative regulators that together negatively regulate TRIF expression, thereby clarifying the role of miRNAs in regulating the host-pathogen interaction networks and providing new insights for future research.

MiRNA inhibits target genes translation or induces mRNA degradation at the posttranscriptional level to achieve the negative regulation of the target protein. Since let-7 was first identified as a miRNA, an increasing number of miRNAs have been discovered. It can inhibit the translation and further inhibit protein expression in nematodes by complementing the 3’UTR region of the target gene, thereby regulating the function of organisms (51). Studies have shown that Stat3 and C/EBPβ synergistically induce miR-21 and miR-181b expression during sepsis (52). Later studies found that STAT3 activates miR-21 and miR-181b-1 through PTEN and CYLD as part of an epigenetic switch that links inflammation to cancer (53). MiR-181b can be targeted in the human cancer cell lines BCL2 (to regulate multidrug resistance) (54). Another study demonstrated the prognostic significance of miR-181b and miR-21 in patients with gastric cancer treated with S-1/oxaliplatin or doxfluridine/oxaliplatin (55). Given the important role of miR-21-1 and miR-181b-2 in human research, we attempted to determine the functions of miR-21-1 and miR-181b-2. We first verified that miR-21-1 and miR-181b-2 inhibit the fluorescent activity of TRIF-3’UTR by using double-fluorescence experiments. Thus, we believe that TRIF may be a new target of miR-21-1 and miR-181b-2. Then, we transfected miiuy croaker MBrC cells with miR-21-1 or miR-181b-2 mimics or inhibitors and then detected TRIF and the expression of cytokines used by qRT-PCR. In this study, we first determined the up-regulation of miR-21-1 and miR-181b-2 expression after LPS stimulation, *V. anguillarum*, or poly(I:C) stimulation or SCRV infection of miiuy croaker. We then predicted the target gene for miR-21-1 and miR-181b-2 and presumed that TRIF is a direct target of these miRNAs. Then we transfected miR-21-1 and miR-181b-2 mimics or inhibitors into miiuy croaker MBrC cells and detected changes in the expression levels of a number of inflammatory cytokines such as IL-8, TNF-α, and Mx1. We found that the expression of inflammatory cytokines was significantly decreased after transfection of miR-21-1 and miR-181b-2, and the expression of inflammatory cytokines was up-regulated after transfection with miR-21-1 and miR-181b-2 inhibitor. We also examined the expression levels of inflammatory cytokines. We verified that miR-21-1 and miR-181b-2 can inhibit the expression of TRIF protein at the translation level and inhibit reporter genes, such as NF-κB, activated by TRIF. The results of this study will improve the understanding on the innate immune response mechanism of fish and provide new insights into the study of mammalian TRIF.

## Data Availability Statement

The original contributions presented in the study are included in the article/**Supplementary Material**. Further inquiries can be directed to the corresponding authors.

## Author Contributions

Conceived and designed the experiments: TX and LH. Performed the experiments: YS, LZ, and WZ. Analyzed the data: YS, LZ, WZ, and JC. Contributed reagents/materials/analysis tools: TX, LH, and XL. Wrote the paper: YS, LZ, WZ, and JC. All authors contributed to the article and approved the submitted version.

## Funding

This study was supported by the National Natural Science Foundation of China (31802325 and 31822057) and Open Fund of CAS Key Laboratory of Experimental Marine Biology, Institute of Oceanology, Chinese Academy of Sciences (KF2019NO1).

## Conflict of Interest

The authors declare that the research was conducted in the absence of any commercial or financial relationships that could be construed as a potential conflict of interest.

## Publisher’s Note

All claims expressed in this article are solely those of the authors and do not necessarily represent those of their affiliated organizations, or those of the publisher, the editors and the reviewers. Any product that may be evaluated in this article, or claim that may be made by its manufacturer, is not guaranteed or endorsed by the publisher.
