# Supplementary material for: MicroRNA-181b-2 and MicroRNA-21-1 Negatively Regulate NF-κB and IRF3-Mediated Innate Immune Responses via Targeting TRIF in Teleost
Source: Front Immunol. 2021 Dec 9;12:734520. doi: 10.3389/fimmu.2021.734520 (PMC8695722; doi:10.3389/fimmu.2021.734520)
Supplement: Supplementary file 1 [file DataSheet_1.pdf]

Supplementary Table 1. PCR primer sequence information in this study.

**Primers**	**Sequences (5'-3')**
**Vector construction**	
pre-miR-181b-2-1F	CCCAAGCTTGCTTGCTCCAACTACGTCAG
pre-miR-181b-2-1R	CCGGAATTCCACATTCACTCCGCTTCCTT
pre-miR-21-1-1F	CCCAAGCTTGGCATACAGACAGGTCG
pre-miR-21-1-1R	CCGGAATTCTTGGAAGAATTGCGTCA
TRIF -3'UTR-1F	CTAGCTAGCGCTGATTGTAGAAATTGTGAGAGGT
TRIF -3'UTR-1R	TGCTCTAGAAAACAAGCATACACACGAGT
TRIF -3'UTR-GFP-1F	CCCAAGCTTGCTGATTGTAGAAATTGTGAGAGGT
TRIF -3'UTR-GFP-1R	CGCGGATCCAAACAAGCATACACACGAGT
TRIF -1F	GACGATGACGACAAGAAGCTTATGAGCCGCGAGGGAGAA
TRIF -1R	CTAAAGACATTGCTCATCTGAATCATC
TRIF -3'UTR-1F	CAGATGAGCAATGTCTTTAGATGTAAAAATATGATGACATATGGTTAGAAT
TRIF -3'UTR-1R	TGATGGATATCTGCAGAATTCAAACAAGCATACACACGAGTTATACAGC
TRIF -3'UTR-MT1-1F	GGCCTTTCATTTGCGGTTTAGCAGAAGAAGACTGCCATCT
TRIF -3'UTR-MT1-1R	AACCGCAAATGAAAGGCCTTGTGCTGCTGTTCA
TRIF -3'UTR-MT2-1F	GAGCAATGAGTTACACGCTCCTTATTGCTGCTC
TRIF -3'UTR-MT2-1R	GCGTGTAACTCATTGCTCCCTAGTGCCTAAAGT
**Real-time PCR**	
TRIF-RT-1F	TGTCCCAATGTGCCACCA
TRIF-RT-1R	TCACCTCCACCCAATCCC
IL-8-RT-1F	AGCAGCAGAGTCTTCGT
IL-8-RT-1R	TCTTCGCAGTGGGAGTT
TNF-α-RT-1F	GTTTGCTTGGTACTGGAATGG
TNF-α-RT-1R	TGTGGGATGATGATCTGGTTG
MX1-RT-1F	GCTGCTTGTTTACTCCCA
MX1-RT-1R	ACCTGCATCATCTCCCTC
β-actin-RT-1F	GTGATGAAGCCCAGAGCA
β-actin-RT-1R	CGACCAGAGGCATACAGG
miR-181b-2-RT-1F	AACATTCATTGCTGTCGCT
miR-181b-2-RT-1R	GTCCAGTTTTTTTTTTTTTTTCCCA
miR-21-1-RT-1F	AGCAACAGCAGTCTGTAAG
miR-21-1-RT-1R	TCCAGTTTTTTTTTTTTTTTGCCA
5.8S rRNA-RT-1F	AACTCTTAGCGGTGGATCA
5.8S rRNA-RT-1R	GTTTTTTTTTTTTTTTGCCGAGTG
