# Supplementary material for: MicroRNA-181b-2 and MicroRNA-21-1 Negatively Regulate NF-κB and IRF3-Mediated Innate Immune Responses via Targeting TRIF in Teleost
Source: Front Immunol. 2021 Dec 9;12:734520. doi: 10.3389/fimmu.2021.734520 (PMC8695722; doi:10.3389/fimmu.2021.734520)
Supplement: Supplementary file 2 [file DataSheet_2.pdf]

**Supplementary Table 2.** The predicted potential miRNAs targeting TRIF

| Gene | Predicted miRNAs targeting TRIF |
| --- | --- |
| TRIF | antisense-miR-16-1-5p |
| | antisense-miR-2300a-1-5p |
| | antisense-miR-3120-1-3p |
| | antisense-miR-3596-2-5p |
| | let-7a-1-5p |
| | let-7d-1-5p |
| | miR-106a-3p |
| | miR-122-5p |
| | miR-125a-1-5p |
| | miR-126-5p |
| | miR-128-1-3p |
| | miR-132-1-3p |
| | miR-142b-1-3p |
| | miR-144-5p |
| | miR-15a-5p |
| | miR-15b-1-3p |
| | miR-15b-1-5p |
| | miR-16-1-5p |
| | miR-16-2-5p |
| | miR-17-1-3p |
| | miR-17-2-3p |
| | miR-181a-1-5p |
| | miR-181b-1-5p |
| | miR-181b-2-5p |
| | miR-183-5p |
| | miR-187-3p |
| | miR-187-5p |
| | miR-194a-5p |
| | miR-194b-5p |
| | miR-196a-5p |
| | miR-196b-3p |
| | miR-196b-5p |
| | miR-199-1-3p |
| | miR-19a-3p |
| | miR-19b-1-3p |
| | miR-19d-3p |
| | miR-200a-3p |
| | miR-200b-3p |
| | miR-20-2-3p |
| | miR-21-1-3p |
| | miR-21-2-3p |
| | miR-216a-2-5p |
| | miR-217-5p |
| | miR-218a-5p |
| | miR-221-3p |
| | miR-222-1-3p |
| | miR-222-2-5p |
